# Supplementary figures and images for: TMPRSS2 promotes SARS-CoV-2 evasion from NCOA7-mediated restriction
Source: PLoS Pathog. 2021 Nov 22;17(11):e1009820. doi: 10.1371/journal.ppat.1009820 (PMC8648102; doi:10.1371/journal.ppat.1009820)

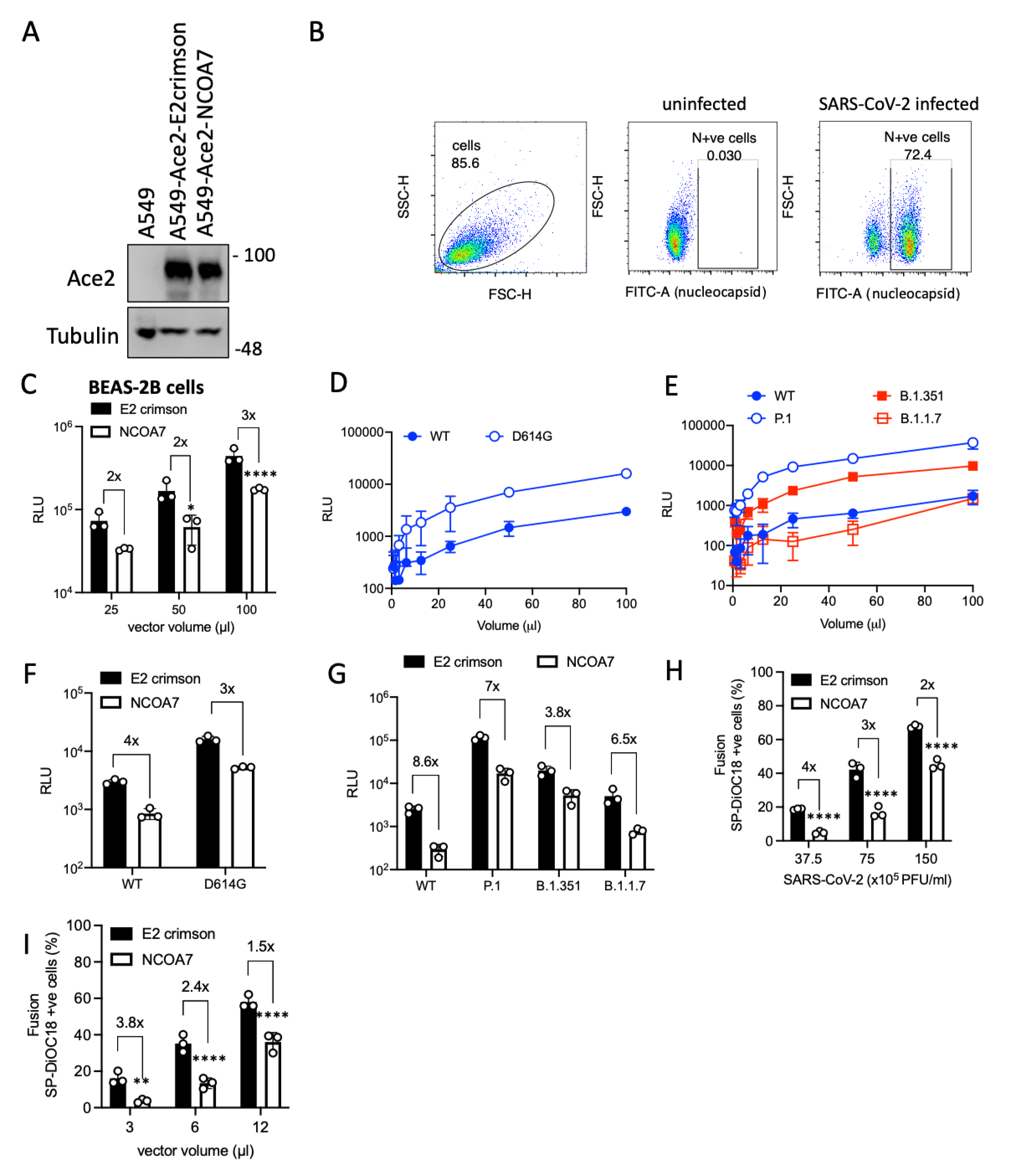

Supplement: S1 Fig — (A) Immunoblot showing ACE2 expression in A549 cells after transduction with ACE2 expressing vector. Tubulin is included as a loading control. (B) Gating strategy for flow cytometry analysis of SARS-CoV-2 nucleocapsid (N) positive cells. Cells were distinguished from debris using the forward scatter (FCS, related to the cell size) and side scatter (SSC, related to the cell granularity). Selected population was gated for N positive cells (FITC channel) using uninfected cells as a negative control. SARS-CoV-2 infected cells were then gated for N. (C) BEAS-2B-ACE2 cells expressing E2crimson or NCOA7 were infected with SARS-CoV-2 Spike pseudotyped vector expressing luciferase. Luciferase activity was measured 48 h post-infection. (D) A549-ACE2 cells were infected with increasing volumes of wild-type (WT) or D614G SARS-CoV-2 Spike pseudotyped vectors expressing luciferase. Luciferase activity was measured 48 h post-infection. (E) A549-ACE2 cells were infected with increasing volumes of wild-type (WT), P.1, B.1.351 or B.1.1.7 SARS-CoV-2 Spike pseudotyped vectors expressing luciferase. Luciferase activity was measured 48 h post-infection. (F) A549-ACE2 cells expressing E2crimson or NCOA7 were infected with wild-type (WT) or D614G SARS-CoV-2 Spike pseudotyped vectors expressing luciferase. Luciferase activity was measured 48 h post-infection. (G) A549-ACE2 cells expressing E2crimson or NCOA7 were infected with wild-type (WT), P.1, B.1.351 or B.1.1.7 SARS-CoV-2 Spike pseudotyped vectors expressing luciferase. Luciferase activity was measured 48 h post-infection. (H) A549-ACE2 cells expressing E2crimson or NCOA7 were infected with SP-DiOC18 labelled SARS-CoV-2 for 1.5 h. SP-DiOC18 positive cells were enumerated by flow cytometry. (I) A549-ACE2 cells expressing E2crimson or NCOA7 were infected with SP-DiOC18 labelled SARS-CoV-2 Spike pseudotyped vector for 1.5 h. SP-DiOC18 positive cells were enumerated by flow cytometry. (C, H, I) analysed using two-way ANOVA. * (p<0.05), ** [file ppat.1009820.s001.tif]
