# Supplementary material for: TMPRSS2 promotes SARS-CoV-2 evasion from NCOA7-mediated restriction
Source: PLoS Pathog. 2021 Nov 22;17(11):e1009820. doi: 10.1371/journal.ppat.1009820 (PMC8648102; doi:10.1371/journal.ppat.1009820)
Supplement: Attachment — Submitted filename: 281021 NCOA7 rebuttal.docx [file ppat.1009820.s002.docx]

We thank all the reviewers for their encouraging and positive comments. We have addressed these point-by-point below (text in red).

Please pay particular attention to central themes across the several reviews:

(1) The lack of any statistics was noted by all four reviewers and must be rectified this age of rigor and reproducibility. For that matter, I agree that a more quantifiable way of measuring virus input is required for cross-comparison across experiments and figures (Reviewer 2).

As requested, statistical tests have now been applied and are described in the figure legends. The vector dose has been normalised according to reverse transcriptase (RT) activity as measured by SG-PERT for Figure1 D & E as suggested by reviewer 2.

(2) At the the end of the day, it is still unclear how the interplay between furin cleavage site and TMPRSS2 activity modulates NCOA7 restriction of SARS2 infection (Reviewers1 and 4). Sone of the data provided appear counter-intuitive. Instead of speculation, I urged the authors to provide data that directly link how enhanced acidification or proteolysis impacts on coronavirus fusion.

To summarise our viewpoint, viral entry that occurs via the endocytic pathway is more sensitive to suppression by NCOA7 than entry occurring at the cell surface. This is attributable to the disruptive effects of NCOA7 on the endocytic pathway. Accordingly, “factors” that promote entry via the endocytic route will sensitise the virus to NCOA7-mediated inhibition, whereas “factors’’ favouring cell surface entry (e.g., TMPRSS2) will have the opposite effect. As suggested, and to substantiate this model, we have therefore performed a lipophilic dye-based fluorescence dequenching assay using SP-DiOC18 to study the direct effect of NCOA7 on SARS-CoV-2 membrane fusion. SARS-CoV-2 pseudotyped and infectious particles were labelled with SP-DiOC18 (the green fluorescence of DiOC18 is suppressed by self-quenching in viral particles). Fluorescence dequenching of SP-DiOC18 occurs following viral–endosome membrane fusion, and this was observed as significant decreases in the number of SP-DiOC18 positive cells in the presence of NCOA7 (Figure S1H, S1I).

(3) Since SARS2 entry into airway epithelial cells expressing ACE2 and TMPRSS2 are most physiologically relevant, the authors should provide data on cell lines accepted to be more physiological mimics of airway epithelial cells (e.g. Calu-3) or primary airway epithelial cells (even if it is for the key experiments to support the authors main point). Concerns from Reviewers 3 and 4 should be taken seriously in this regard.

Thank you for this helpful suggestion. We have now tested the activity of NCOA7 in a human bronchiolar epithelial cell line, BEAS-2B, which has been utilized as an infection model for several respiratory viruses. The data are shown in supplementary Figure S1C.

**Part I - Summary**
Please use this section to discuss strengths/weaknesses of study, novelty/significance, general execution and scholarship.

**Reviewer #1:** Previously, the authors identified an interferon (IFN) inducible gene, NCOA7, that inhibits entry of viruses via the endo-lysosome pathway. SARS-CoV-2 primarily enters host cells by fusing with the plasma membrane wherein transmembrane serine protease TMPRSS2 processes the viral spike protein to facilitate viral entry (1, 2). However, SARS-CoV-2 can also enter cells through the endo-lysosome pathway where cathepsins process the spike protein (2). Using lung epithelial A549 cells expressing the ACE2 receptor, the authors show that in the absence of TMPRSS2, overexpressing NCOA7 decreases SARS-CoV-2 infection. However, in the presence of TMPRSS2, the inhibition mediated by NCOA7 is attenuated. The authors conclude NCOA7 controls SARS-CoV-2 infection only when the virus enters the cell through the endo-lysosomal pathway. It was noted that in comparison to SARS-CoV-2, NCOA7 restriction of infection was not as pronounced with SARS-CoV-1. A major difference between the two viruses is the presence of a polybasic furin cleavage site in SARS-CoV-2 which enables initial processing of the spike protein in the Golgi network during virus production (2). Therefore, the authors investigated the role of the furin cleavage site in NCOA7 mediated restriction. Deletion of the furin cleavage site in SARS-CoV-2 decreased NCOA7 restriction. Conversely, addition of a furin cleavage site to SARS-CoV-1 enhanced NCOA7 restriction of the virus. The authors suggest the furin cleavage site sensitizes SARS-CoV-2 to NCOA7 inhibition within the endo-lysosomal pathway so the virus preferentially exploits TMPRSS2 to enter the host cell at the plasma membrane.

1. V’kovski, P., Kratzel, A., Steiner, S. et al. Coronavirus biology and replication: implications for SARS-CoV-2. Nat Rev Microbiol 19, 155–170 (2021).
2. Peacock, T.P., Goldhill, D.H., Zhou, J. et al. The furin cleavage site in the SARS-CoV-2 spike protein is required for transmission in ferrets. Nat Microbiol 6, 899–909 (2021).
3. Hoffmann, M., Kleine-Weber, H., Pöhlmann, S. A Multibasic Cleavage Site in the Spike Protein of SARS-CoV-2 Is Essential for Infection of Human Lung Cells. Mol Cell 78, 779-784 (2020).
4. Johnson, B.A., Xie, X., Bailey, A.L. et al. Loss of furin cleavage site attenuates SARS-CoV-2 pathogenesis. Nature 591, 293–299 (2021).
5. Lei, X., Dong, X., Ma, R. et al. Activation and evasion of type I interferon responses by SARS-CoV-2. Nat Commun 11, 3810 (2020).

**Reviewer #2:** In this work, the authors test the role of NCOA7 broadly for restriction of human coronaviruses. Despite its broad efficacy, the overexpression of TMPRSS2 or removal of the spike poly-basic cleavage site can help to avoid this restriction. The authors suggest that viral acquisition of a furin cleavage site and use of the co-receptor may have been selected for to avoid the suppressive effects of interferon. Understanding restriction of coronaviruses is an important topic and this study is well suited for this journal. While the experiments appear to be well-performed from a technical perspective, the lack of statistical analysis makes many of the conclusions drawn by the authors difficult to evaluate. Further, previous work on this restriction factor would allow one to predict with high confidence the results of this study, potentially limiting the impact of this work. My specific comments are below.

**Reviewer #3:** This is an interesting study of an interferon-stimulated gene and its potential role as a restriction factor for SARS-CoV-2 and other coronaviruses. NCOA7 acts within the endolysosomal pathway and would thus be expected to affect pseudovirus entry only when a) SARS-CoV-2 enters via that route or b) if somehow endolysosomal trafficking alters cell-surface expression of other entry factors. The major weakness of this study is its reliance on the A549 cell line, which is not a preferred model for SARS-CoV-2 infection. The results should be compared with NCOA7 effects on entry in Calu-3 cells or primary human respiratory epithelial cells. One might anticipate that the effects could be attenuated in Calu-3 because of the increased effect of TMPRSS2 in those cells, which the authors note reduces the NCOA7 effects observed.

Broadly, this line of inquiry would be a useful addition to the literature. The major weaknesses of both significance and rigor come from limiting the experiments to a cell type that is not believed to reproduce key properties for SARS-CoV-2 entry.

**Reviewer #4:** Previous studies have identified two cellular entry pathways for SARS-CoV-2: a mechanism involving endocytosis, cathepsin-mediated cleavage of the viral spike protein (S) and fusion with endosome membranes occurs in many cell lines, whereas cell surface fusion triggered by the serine protease TMPRSS2 has been suggested to be the main mechanism for entry into epithelial cells in organoid models of the respiratory epithelium. The authors of this paper have previously identified an interferon inducible protein NCOA7 that restricts the entry of pH-dependent viruses that infect cells via endocytic routes. NCOA7 interacts with the v-ATPase responsible for the acidification of endocytic organelles and appears to enhance both acidification and the activity of lysosomal hydrolases. In this paper, the authors show that NCOA7 also antagonises SARS-CoV-2 (and other coronaviruses known to infect humans) when virus entry is restricted to the endocytic route. The work is topical and likely to be of interest to not only SARS-CoV-2 researchers, but to virologists and cell biologists in general. However, I do have a few concerns the authors should consider.

Using primarily a respiratory epithelial derived cell line, A549, the authors show that NCOA7 expression inhibits infection by SARS-CoV-2 and by pseudotype viruses expressing SARS-CoV-2 S; they show that interferon-alpha treated cells knocked out for NCOA7 are less well protected than wt cells. They also show that other human coronaviruses and pseudotype viruses expressing S proteins from SARS-CoV-2 variants of concern are similarly affected, and that expression of TMPRSS2 or deletion of the polybasic furin cleavage site reduces NCOA7 mediated inhibition of infection by 2-3 fold. Together, the data make a compelling case for NCOA7 being an interferon inducible antagonist of coronavirus entry via the endocytic route.

**Part II – Major Issues: Key Experiments Required for Acceptance**
Please use this section to detail the key new experiments or modifications of existing experiments that should be absolutely required to validate study conclusions.

Generally, there should be no more than 3 such required experiments or major modifications for a "Major Revision" recommendation. If more than 3 experiments are necessary to validate the study conclusions, then you are encouraged to recommend "Reject".

**Reviewer #1:**

1. Mechanistically, it is unclear how the presence of a furin cleavage site enhances NCOA7 restriction of SARS-CoV-2. Acquisition of the furin cleavage site is believed to be directly correlated with increased virus replication pathogenicity (3,4). If the furin cleavage site is negatively affecting the virus as the authors propose, why does the virus maintain this polybasic site?

Thank you for this comment. In this manuscript, we show that the polybasic furin cleavage site negatively affects virus entry in cells expressing very little or no TMPRSS2. In such cells, the majority of fusion occurs in endosomes and is therefore sensitive to NCOA7 expression. The use of TMPRSS2 or other cell surface proteases is thought to promote pH-independent fusion at the cell surface, thus avoiding restriction by antiviral mechanisms operating in the endocytic pathway (e.g., NCOA7). Thus, the furin cleavage site promotes pathogenicity, and, we hypothesize, is enabled to do so by the exploitation of cell surface proteases such as TMPRSS2.

Nevertheless, the precise sites of viral-membrane fusion under different conditions deserve further detailed attention. Accordingly, though beyond the scope of this study, we are pursuing single-virus tracking experiments in live-cells to dissect the precise mechanism of how the presence of a furin cleavage site enhances NCOA7 restriction of SARS-CoV-2, and is counteracted by TMPRSS2.

2. SARS-CoV-2 encodes mechanisms to block and delay IFN production (5). What is the biological significance of IFN inducible NCOA7 in cells that do not express TMPRSS2? Do these cells produce IFN early enough during infection to prevent viral entry?

SARS‐CoV‐2 has been shown to trigger a robust innate immune response through the activation of cytoplasmic RNA sensors, RIG‐I and MDA5, in lung epithelial cells. However, IFN production is delayed and unable to suppress infection during an *in vitro* challenge. We have not measured IFN induction in cells that do not express TMPRSS2 but we have tested the effect of endogenous NCOA7 after adding exogenous IFN in Figure 1F and G to show that in the absence of NCOA7 the inhibitory effect of IFN is diminished.

3. Statistical analyses are lacking in all figures.

Statistical tests have now been applied throughout.

**Reviewer #2:**

1. For all figures, it does not appear that any statistical analysis was ever performed, but in some cases the fold-change is displayed. The appropriate statistical analyses should be applied whenever a comparison is being drawn. Without it, it is almost impossible for a reader to determine if potential changes are meaningful (for example Fig 1G or H).

Statistical tests have now been applied throughout.

2. For many figures, the x-axis describes "Vector volume" to indicate the magnitude of the pseudo typed virus inoculum. While I understand this is convenient, it makes it difficult to compare between viruses. And in some cases, this concern is important evaluate the author's conclusions. For example, Figure 1D and E, we are expected to conclude that NCOA7 restricts SARS-2 but not MLV, however the pseudo virus packaging could have been much more efficient for MLV and a dramatically higher "MOI" could overwhelm restriction. A more standardized quantification such as p24 ELISA would help alleviate this concern.

Thank you for the suggestion. For Figure 1D and E, the vector dose has now been normalised by RT activity as measured by SG-PERT. For all the comparisons made between vectors, the vectors were produced at the same time (which generally produces equivalent viral yields); and, in addition, the use of multiple input doses in the majority of the experiments ensured that measurements of infection were made in the quantitative range where differences were not “masked” by inocula being either too high or too low. This is now described more explicitly in the Methods.

3. Figure 3: The cross-experiment comparisons for panels C, D and E, F are not convincing. These comparisons must be done within the same experiment (side-by-side) for a reader to appreciate if the difference between a 4X change in the absence of TMPRSS2 and a 2X change with TMPRSS2 is meaningful or the result of variability between experiments.

The reviewer is correct, and we apologise that this was not clear. The experiments shown in Figure 3C-D and 3E-F were done in parallel (side-by-side) to make comparisons. We have now clarified this by putting the data with or without TMPRSS2 together as Figure 3C (SARS-2) and Figure 3D (SARS-1).

4. SARS-CoV-2 has been demonstrated to not induce strong interferon responses; this seems at odds with the authors suggestion that IFN-induced NCOA7 may have been a strong selection pressure on the virus. This should be discussed.

This may be true, but the IFN response is important in the context of natural infection, as illustrated, inter alia, through the study of individuals with inborn errors in the interferon system. The interplay between SARS-CoV-2 and innate immunity/interferon is undoubtedly very complex, and will require extensive investigation before the many facets are understood. In this manuscript, we have provided evidence that one particular ISG (NCOA7) can inhibit SARS-CoV-2, that its effect is seen in the presence of interferon, and that TMPRSS2 can help evade the inhibitory effect.

**Reviewer #3:**

1. Repeat results in Calu-3 or primary human respiratory epithelial cultures

Thank you for the suggestion. We have now tested the activity of NCOA7 in a human bronchiolar epithelial cell line, BEAS-2B, which has been utilized as a model for several respiratory virus infections (1–4). The data are shown in supplementary Figure S1C.

1.	Hillyer P, Shepard R, Uehling M, Krenz M, Sheikh F, Thayer KR, et al. Differential Responses by Human Respiratory Epithelial Cell Lines to Respiratory Syncytial Virus Reflect Distinct Patterns of Infection Control. J Virol [Internet]. 2018 Jul 17;92(15):e02202-17. Available from: https://pubmed.ncbi.nlm.nih.gov/29769339

2.	Gan H, Hao Q, Idell S, Tang H. Transcription Factor Runx3 Is Induced by Influenza A Virus and Double-Strand RNA and Mediates Airway Epithelial Cell Apoptosis. Sci Rep [Internet]. 2015;5(1):17916. Available from: https://doi.org/10.1038/srep17916

3.	Brandao-Rangel MAR, Melamed D, Silva-Reis A, Brill B, Santos Zamarioli L dos, Oliveira CR, et al. Virlaza^TM^ Inhibits Sars-COV-2-induced Inflammatory Response of Bronchial Epithelial Cells and Pulmonary Fibroblast. medRxiv [Internet]. 2021 Jan 1;2021.07.17.21260123. Available from: http://medrxiv.org/content/early/2021/07/22/2021.07.17.21260123.abstract

4.	Wang S, Qiu Z, Hou Y, Deng X, Xu W, Zheng T, et al. AXL is a candidate receptor for SARS-CoV-2 that promotes infection of pulmonary and bronchial epithelial cells. Cell Res. 2021;31(2):126–40. Available from: https://doi.org/10.1038/s41422-020-00460-y

**Reviewer #4:**

1. One issue I have with this work is the mechanism of NCOA7 action. To me it seems somewhat counter intuitive that a protein that enhances acidification and lysosomal activity should inhibit entry of a virus that relies on these activities to trigger fusion. The authors argue that excess or premature acidification negatively affects the ability of the virus to undergo fusion. While this may be true for influenza virus, as indicated in their previous paper, the key event for SARS-CoV-2 fusion is proteolytic cleavage of S, and there is no data in this paper to indicate that enhanced acidification/proteolysis impacts on coronavirus fusion.

Thank you for this constructive comment. In responding, we have now performed a lipophilic dye-based fluorescence dequenching assay using SP-DiOC18 to study the direct effect of NCOA7 mediated endosomal acidification on SARS-CoV-2 membrane fusion. As shown in Figure S1H and S1I, NCOA7 expression reduces the extent of dequenching in challenges with pseudotypes and infectious SARS-CoV-2, consistent with diminished efficiency of viral membrane fusion.

2. Secondly, the authors argue that the presence of the furin cleavage site may have allowed the virus to use TMPRSS2 and a cell surface route of entry to evade NCOA7-mediated restriction. It seems to me that the virus without a furin cleavage site can still infect cells by a TMPRSS2 dependent route (Figs 3F and 5), albeit less efficiently. What the furin cleavage site may do is allow more efficient TMPRSS2-mediated activation of S. It’s worth noting that TMPRSS2 expression also reduces NCOA7 inhibition of SARS-1 infection – a virus that lacks the polybasic furin cleavage site? Polybasic cleavage sites do occur in other viruses (e.g. flu), with no obvious impact on the site and underlying mechanism of fusion, as far as I am aware, i.e. for flu at least, there is no evidence the acquisition of a furin cleavage site is linked to escape from NCOA7 mediate restriction.

Thank you for making these important points.

We agree with the reviewer that the furin cleavage site enhances the efficiency of TMPRSS2 mediated cleavage. We point the reviewer to lines 291-294 in the discussion.

It is interesting that TMPRSS2 expression reduces NCOA7 inhibition of SARS-CoV-1 which lacks a furin cleavage site in Spike. However, TMPRSS2 has been shown to cleave SARS-CoV-1 Spike and reduce the requirement for acidic pH and cathepsin activity for SARS-CoV-1 Spike driven entry (1). Furthermore, trypsin which cleaves the SARS-CoV-1 Spike at the same site as TMPRSS2 allows evasion from IFITM mediated restriction of SARS-CoV-1 suggesting pH independent fusion at the plasma membrane (2).

1. Glowacka I, Bertram S, Müller MA, et al. Evidence that TMPRSS2 activates the severe acute respiratory syndrome coronavirus spike protein for membrane fusion and reduces viral control by the humoral immune response. *J Virol*. 2011;85(9):4122-4134. doi:10.1128/JVI.02232-10
2. Huang IC, Bailey CC, Weyer JL, et al. Distinct patterns of IFITM-mediated restriction of filoviruses, SARS coronavirus, and influenza A virus. *PLoS Pathogen*. 2011;7(1):e1001258. Published 2011 Jan 6. doi:10.1371/journal.ppat.1001258

The situation with furin cleavage site acquisition in influenza virus is different to SARS-CoV-2. In the former, the site of cleavage in HA remains unchanged, but, rather, it is the range of proteases that can cleave that site that changes. For SARS-CoV-2, a new cleavage site is introduced, leading to the changes in Spike function discussed herein and elsewhere. Accordingly, we do not have immediate plans to examine the consequences of furin cleavage site acquisition in influenza virus HA with regards to NCOA7 function.

3. As entry into respiratory epithelial cells in organoid systems, seems to occur primarily by TMPRSS2-mediated cell surface fusion, does NCOA7 play any role in controlling virus infection and spread in primary tissues, in particular the respiratory epithelium?

This is an important question for future investigation and is beyond the scope of this manuscript. Protein overexpression or depletion in primary respiratory tissues does not work very well, however, we are keen to collaborate with experts in the culture of respiratory epithelium or relevant iPSC-derived systems to investigate this further. In this work we have examined the role of NCOA7 in two respiratory epithelial cell lines, A549 and BEAS-2B which are widely used for studying respiratory infections.

**Part III – Minor Issues: Editorial and Data Presentation Modifications**
Please use this section for editorial suggestions as well as relatively minor modifications of existing data that would enhance clarity.

**Reviewer #1:**

1. In figure 4, relative light units (RLUs) for control samples are vastly different. For example, RLUs in figure 4B for A549-ACE2 untreated cells without TMPRSS2 are slightly above 103. In figure 4C, the same conditions (A549-ACE2 untreated cells without TMPRSS2) yield 104 RLUs.

In figure 4, no comparison is being made between the different figures so the variation in RLUs does not undermine the conclusions drawn from the data. The variation in RLUs seen in figure 4B and 4C is because the experiments were done on different days with a different batch of the vectors.

2. In figures 1F and 1E, are the cells being treated with IFNβ or IFNα? The figure axes are labeled IFNβ, but the figure legends state the cells are treated with IFNα.

Thank you for pointing out this error. The figures have been corrected. IFNβ in figure 1F and 1E has been replaced with IFNα.

**Reviewer #2:**

1. Figure 5E, the text is non-visible in the figure legends, I assume the authors are indicating microliters. Same comment for Sup Fig 1 C,D

Text size has been increased for these figures.

**Reviewer #3:**

1. Biosafety precautions for replication-competent SARS-CoV-2 should be stated explicitly.

The following statement has been added to the Replication competent SARS-CoV-2 section in methods. “All work with infectious SARS-CoV-2 was carried out in a Containment Level 3 facility (Health and Safety Executive approvals, CBA1.295.20.1 and GM386/20.2).”

2. "Each experiment shown is representative of three independent experiments." - if this statement were literally true, it would be unacceptable. However, it appears that each experiment was performed in triplicate with the results of each plotted. If such is correct, please alter the statement to reflect this.

The statement has been altered to reflect that “Each figure shown is from three independent experiments”.

**Reviewer #4:**

1. Given MLV also requires endocytosis for entry, though perhaps not acidification, and that NCOA7 increases lysosomal degradation, is it not a bit surprising that NCOA7 expression has no effect of MLV entry? Or is there a small effect? NB, Rasmussen I, Vilhardt F. describe the entry of MLV as macropinocytosis, not micropinocytosis.

Thank you for correcting this error: micropinocytosis has been changed to macropinocytosis. Regarding the result with amphotropic MLV Env, the lack of sensitivity to NCOA7 maybe of interest, but is a consistent finding in different cells lines. We plan to follow-up on this point in future live cell and single-virus tracking experiments.

2. Line 145. Can the authors speculate on why is B.1.351 S protein pseudotypes are less sensitive to NCOA7? Is there any evidence this is giving this VOC an advantage?

This is an interesting point, but at this time we do not have any data that shed light on the mechanistic basis for this. Again, future work will seek to understand the underlying basis for strain specific variation in NCOA7 sensitivity.

3. Why is there still a 2-3 fold effect of NCOA7 on SARS-2 infection in TMPRSS2 expressing cells? Does NCOA7 expression affect ACE2 and/or TMPRSS2 cell surface expression?

These are important questions. First, NCOA7 does not affect ACE2 or TMPRSS2 expression, as demonstrated in the immunoblot included below. Second, regarding the 2-3 fold effect in the presence of TMPRSS2, we assume that a fraction of virus still enters the cell following endocytic events even in the presence of TMPRSS2 expression at the cell surface. In other words, TMPRSS2 promotes entry at the cell surface, but does not ablate entry from the endocytic pathway. Future living cell and single-virus tracking experiments should provide further insight into this point.

4. Do BafA, Niclosamide and/or E64D inhibit the effects of NCOA7?

The exact mechanism by which NCOA7 regulates v-ATPase activity is unclear. However, we found that E64D and BafilomycinA1, which inhibit the activity of cathepsins and v-ATPase respectively, have mildly higher IC50 in the presence of NCOA7.

5. Fig 5. The authors should show that the addition and removal of the polybasic cleavage site does impact on S protein cleavage in virus, or pseudotype, particles.

Thank you for the suggestion. The Spike constructs used in this study were described previously in the publication cited below. The impact of addition or removal of the polybasic cleavage site in Spike was determined by immunoblot.

Winstone H, Lista MJ, Reid AC, Bouton C, Pickering S, Galao RP, et al. The polybasic cleavage site in the SARS-CoV-2 spike modulates viral sensitivity to Type I interferon and IFITM2. J Virol. 2021 Feb 9;95(9):JVI.02422-20.

6. The authors should cite recent papers from the Haagmans lab.

Thank you for the suggestions, the following papers from the Haagmans lab have added to the text.

1. Mykytyn AZ, Breugem TI, Riesebosch S, Schipper D, van den Doel PB, Rottier RJ, Lamers MM, Haagmans BL. SARS-CoV-2 entry into human airway organoids is serine protease-mediated and facilitated by the multibasic cleavage site. Elife. 2021 Jan 4;10:e64508. doi: 10.7554/eLife.64508. PMID: 33393462; PMCID: PMC7806259.
2. Lamers MM, Mykytyn AZ, Breugem TI, Wang Y, Wu DC, Riesebosch S, van den Doel PB, Schipper D, Bestebroer T, Wu NC, Haagmans BL. Human airway cells prevent SARS-CoV-2 multibasic cleavage site cell culture adaptation. Elife. 2021 Apr 9;10:e66815. doi: 10.7554/eLife.66815. PMID: 33835028; PMCID: PMC8131099.

7. A number of symbols in the figures need to be corrected (e.g. Fig . 1 F and G, X axis).

Thank you for pointing this out. The symbols have been corrected.
